# Reduced graphene oxide supported C_3_N_4_ nanoflakes and quantum dots as metal-free catalysts for visible light assisted CO_2_ reduction

**DOI:** 10.3762/bjnano.10.44

**Published:** 2019-02-13

**Authors:** Md Rakibuddin, Haekyoung Kim

**Affiliations:** 1 School of Materials Science and Engineering, Yeungnam University, Gyeongsan, Republic of Korea

**Keywords:** CO_2_ reduction, metal-free hybrid, nanoflakes, photocatalyst, quantum dots

## Abstract

The visible light photocatalytic reduction of CO_2_ to fuel is crucial for the sustainable development of energy resources. In our present work, we report the synthesis of novel reduced graphene oxide (rGO)-supported C_3_N_4_ nanoflake (NF) and quantum dot (QD) hybrid materials (GCN) for visible light induced reduction of CO_2_. The C_3_N_4_ NFs and QDs are prepared by acid treatment of C_3_N_4_ nanosheets followed by ultrasonication and hydrothermal heating at 130–190 °C for 5−20 h. It is observed that hydrothermal exposure of acid-treated graphitic carbon nitride (g-C_3_N_4_) nanosheets at low temperature generated larger NFs, whereas QDs are formed at higher temperatures. The formation of GCN hybrid materials was confirmed by powder X-ray diffraction, X-ray photoelectron spectroscopy, Fourier transform infrared spectroscopy, field emission scanning electron microscopy, transmission electron microscopy (TEM), and UV–vis spectroscopy. High-resolution TEM images clearly show that C_3_N_4_ QDs (average diameter of 2–3 nm) and NFs (≈20–45 nm) are distributed on the rGO surface within the GCN hybrid material. Among the as-prepared GCN hybrid materials, GCN-5 QDs exhibit excellent CO_2_ reductive activity for the generation of formaldehyde, HCHO (10.3 mmol h^−1^ g^−1^). Therefore, utilization of metal-free carbon-based GCN hybrid materials could be very promising for CO_2_ photoreduction because of their excellent activity and environmental sustainability.

## Introduction

The solar-light-assisted photocatalytic reduction of CO_2_ into useful chemicals, such as HCOOH, HCHO, CH_4_, and CH_3_OH is one of the sustainable ways to address the issues of both global warming and the energy crisis [1–6]. So far, a variety of semiconductor photocatalysts, such as ZnO, TiO_2_, WO_3_, and CdS have been developed for the photoreduction of CO_2_ [7–10]. However, poor separation of photo-induced electron–hole pairs and insufficient adsorption of CO_2_ at the catalyst surface are crucial problems preventing effective catalyst performance and CO_2_ reduction [11]. An ideal photocatalyst for CO_2_ conversion should possess a narrow bandgap and good light-harvesting properties, proper conduction band (CB) and valence band (VB) edge positions, exhibit efficient charge separation, have a large surface area, and it must be cost effective. Considering the above factors, nontoxic metal-free catalysts, such as graphitic carbon nitride (g-C_3_N_4_) and reduced graphene oxide (rGO) have received wide attention in recent years for CO_2_ reduction and water splitting applications [12–17]. Both g-C_3_N_4_ and rGO have a two-dimensional sheet structure with high surface area and possess appropriate band edges for CO_2_ reduction. Also, g-C_3_N_4_ and rGO are inexpensive and easy to synthesize.

Despite all these interesting properties, pure g-C_3_N_4_ only weakly absorbs visible light due to its wide band gap and also has poor electrical conductivity [18]. An efficient way to increase the charge separation and electrical conductivity of g-C_3_N_4_ is to modify it with rGO. Besides the structural and electronic modification of the g-C_3_N_4_ material with rGO, another interesting strategy is to increase the number of catalytic active sites (pyridinic N, graphitic N, and edge amine groups) in g-C_3_N_4_ [19]. This can be achieved by generating zero-dimensional (0D) quantum dots (QDs) and nanoflakes (NFs) of g-C_3_N_4_ from 2D sheets by facile hydrothermal reactions. Recently, solar energy conversion using g-C_3_N_4_ QDs has attracted significant attention [20–22]. Besides, g-C_3_N_4_ has also been coupled with noble-metal-free compounds for higher catalytic activity [23–27]. Despite all these significant findings, there have been few studies focused on the improvement of the visible light absorption of g-C_3_N_4_/rGO hybrid materials [28–31] and g-C_3_N_4_ QDs, and their CO_2_ photoreduction ability has not yet been reported.

Hence, in our present study, metal-free hybrid catalysts consisting of rGO-supported C_3_N_4_ (GCN) NFs and QDs are prepared by a hydrothermal method. The formation of the GCN hybrid is controlled over temperature during hydrothermal heating. The as-synthesized GCN hybrids are then characterized and applied to the photoreduction of CO_2_ under visible light. The concentration of the photocatalytic product, formaldehyde (HCHO), is monitored spectrophotometrically using the Nash reagent, and also confirmed by gas chromatography.

## Results and Discussion

### Material characterization

**Figure 1:**
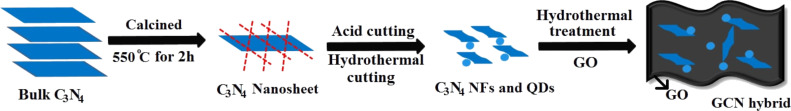
Schematic representation of the preparation of C_3_N_4_ nanoflakes (NFs), quantum dots (QDs) and the rGO-supported C_3_N_4_ (GCN) hybrid material.

**Figure 2:**
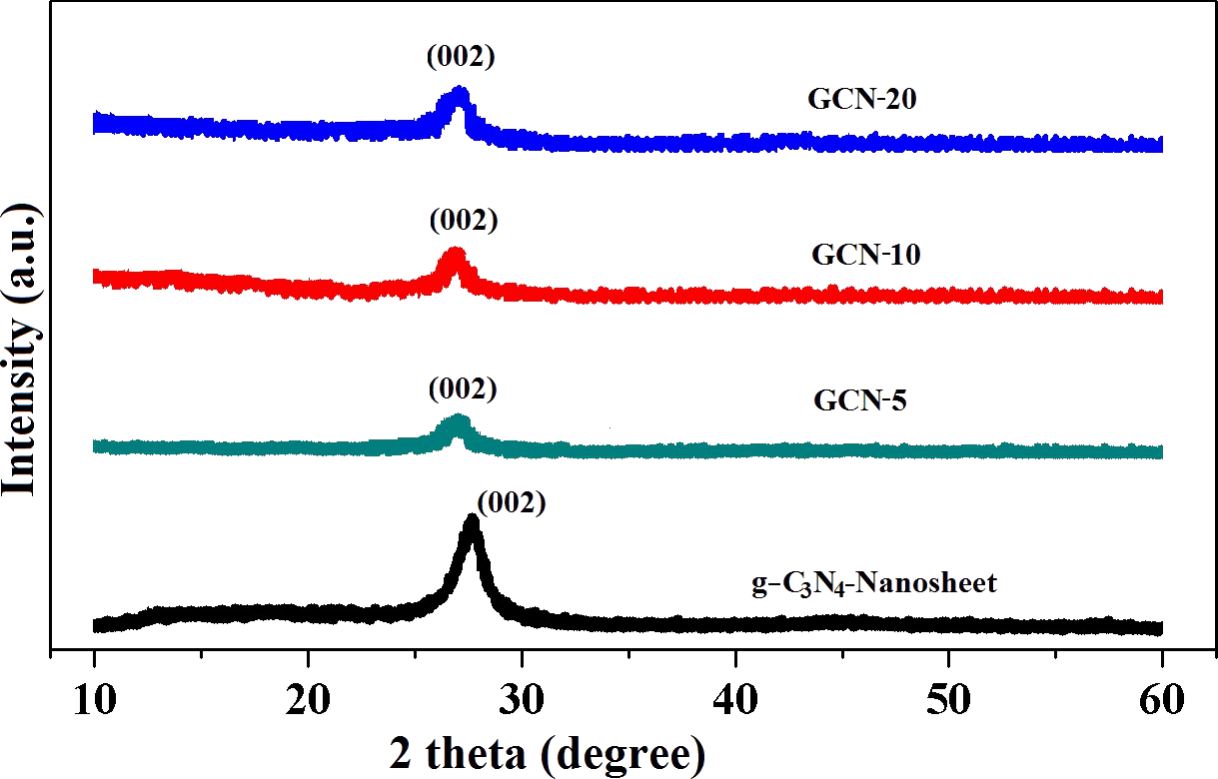
Powder X-ray diffraction patterns of the synthesized rGO-supported C_3_N_4_ (GCN) hybrid materials.

The preparation of g-C_3_N_4_ NFs and QDs from nanosheets and the formation of the GCN hybrid material are shown schematically in Figure 1. When GO is subjected to hydrothermal treatment with CN NFs and QDs, GO converts to rGO (Supporting Information File 1, Figure S1) and GCN hybrid materials are formed. The phase purity of the synthesized g-C_3_N_4_ nanosheet, GCN-5, GCN-10, and GCN-20 hybrid materials are first examined by powder X-ray diffraction (PXRD). Figure 2 shows the PXRD patterns of pure g-C_3_N_4_ nanosheets and GCN hybrids. A broad peak at 2θ = 27.5° is observed for the g-C_3_N_4_ nanosheets, which is due to the interplanar stacking (002 plane) of aromatic C–N heterocycles present in g-C_3_N_4_ [32]. However, the intensity of both (100) and (002) peaks is tremendously decreased for the nanosheets compared to the bulk C_3_N_4_ and is also found to be shifted to a lower angle (Supporting Information File 1, Figure S2). For the GCN hybrid materials, the intensity of the (002) peak of g-C_3_N_4_ nanosheets is significantly decreased. The shifting and decrease in intensity of the (002) peak indicates structural changes of the 2D g-C_3_N_4_ nanosheets to 1D NFs and 0D QDs. The XRD peak intensity of a plane is largely dependent on its internal structure. When the nanosheet transforms into QDs/NFs by acid cutting and hydrothermal heating, it undergoes many structural changes (breaking of aromatic planes along (002) directions) internally. Thus, the size of the nanosheet is drastically decreased when it transforms into quantum dots. Consequently, the intensity of the planes is expected to largely decrease. In the GCN hybrid material, the peaks related to rGO are not observed in the hybrid materials, which may be due to the low amount of rGO incorporation.

**Figure 3:**
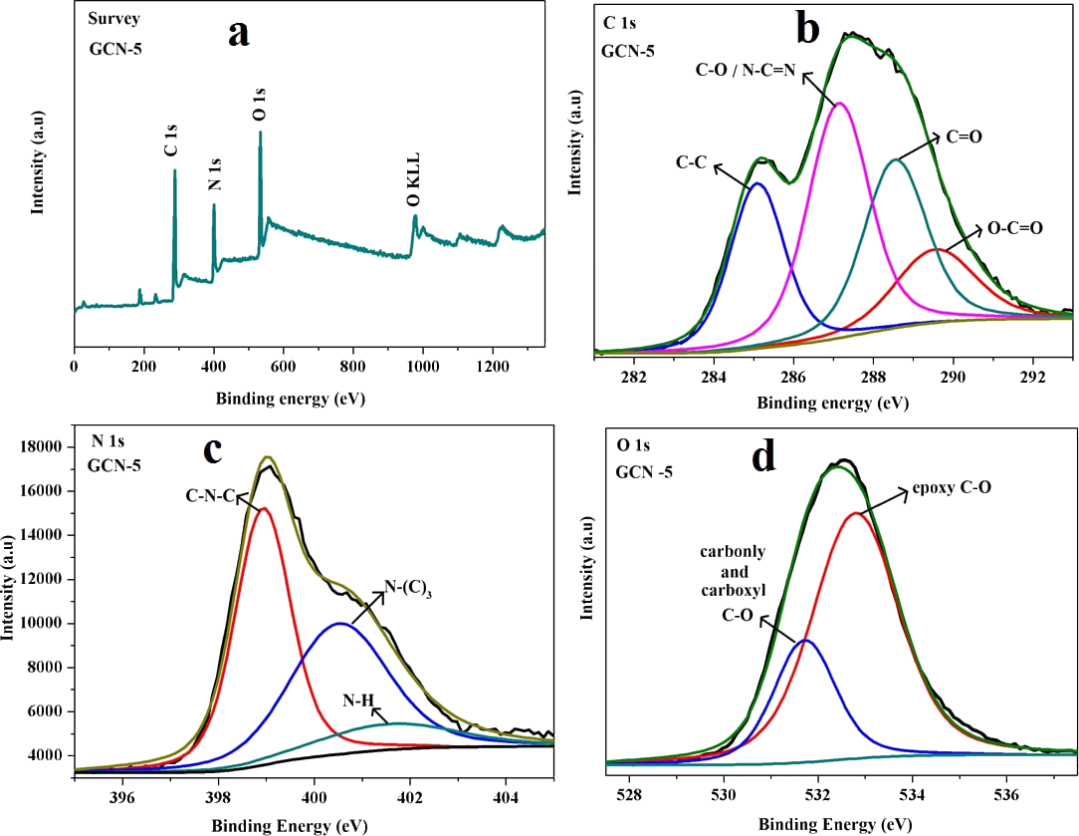
X-ray photoelectron spectroscopy analysis of the prepared GCN-5: a) survey spectrum, and high-resolution b) C 1s, c) N 1s and d) O 1s spectra.

Moreover, the peaks related to other impurities are not found in the pattern, indicating the formation of pure hybrid composite materials. The presence of rGO is confirmed by X-ray photoelectron spectroscopy (XPS) analysis, which is carried out to study the surface composition and the interactions and valence states of the elements present in the hybrid materials. The full XPS survey spectrum of the GCN-5 hybrid material reveals the presence of C, N and O, while no impurity elements are found (Figure 3a). Figure 3b shows the fit to the C 1s peak of the GCN-5 hybrid. The fitting of the C 1s spectrum shows four major deconvoluted peaks related to the carbon states of rGO and g-C_3_N_4_. The sharp peaks at binding energies of 285.04, 287.03, 288.52, and 289.6 eV observed in the C 1s spectrum correspond to C–C bonds in rGO, N–C=N/C–O bonds in g-C_3_N_4_ and rGO, and C=O and O−C=O bonds in rGO, respectively [33–34]. The core-level N 1s profile shows (Figure 3c) three deconvoluted peaks at binding energies of 398.8, 400.6, and 401.8 eV, which are attributed to the sp^2^-hybridized N (C–N=C), the tertiary N, and the N–H group of g-C_3_N_4_, respectively, which are present in the GCN-5 hybrid material [35].

Interestingly, the N peak intensities are significantly decreased compared to those of the pure g-C_3_N_4_ nanosheet (Supporting Information File 1, Figure S3), which is due to the breaking of most C–N bonds due to formation of NFs and QDs. Figure 3d shows a high-resolution XPS spectrum of O 1s present in the GCN-5 hybrid. The peaks at binding energies of 531.7 and 532.8 eV are attributed to carbonyl and epoxy C–O groups of rGO, respectively, which are still present in the rGO [34]. The XPS peaks of g-C_3_N_4_ and rGO are shifted slightly to higher and lower binding energies in the GCN-5 hybrid, respectively, indicating possible charge transfer between g-C_3_N_4_ and rGO in the heterostructure. Hence, the XPS results confirm the successful preparation of the composite and the existence of an interaction between rGO and g-C_3_N_4_ inside the composite.

**Figure 4:**
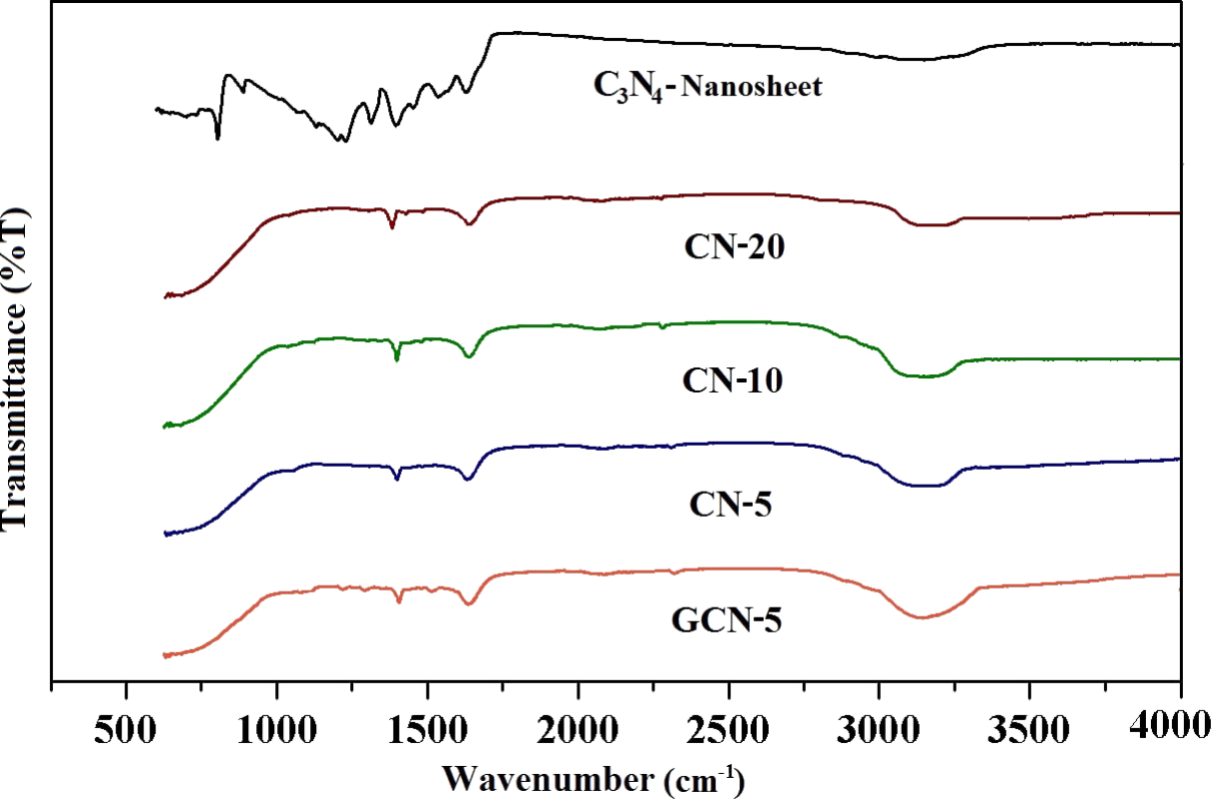
Fourier transform infrared spectra of the prepared g-C_3_N_4_, CN nanoflakes and quantum dots, and GCN-5 photocatalysts.

FTIR spectra further confirm (Figure 4) the formation of C_3_N_4_ NFs and QDs, as well as the structural changes of C_3_N_4_ nanosheets. The peaks at around 3000–3110 cm^−1^, 1200–1650 cm^−1^ and 810 cm^−1^ are due to the N–H stretching vibration, aromatic CN heterocycles, and the s-triazine ring of the g-C_3_N_4_ nanosheet, respectively [36–37]. However, after acid treatment and hydrothermal heating, most of the peaks attributed to CN heterocycles have vanished, indicating the structural transformation of g-C_3_N_4_ nanosheets to C_3_N_4_ NFs and QDs. Besides, a sharp peak at 1380 cm^−1^ is observed, which can be ascribed to –C–O stretching vibrations of carboxylate groups, which are formed due to the breaking of some C–N bonds of triazine rings and their oxidation to carboxylic groups in the C_3_N_4_ NFs and QDs [19]. After the introduction of rGO to form GCN-5, the characteristic peaks of C_3_N_4_ NFs and QDs (CN-5, CN-10, and CN-20) could still be observed along with some smaller intense peaks (epoxy group, 1150–1250 cm^−1^) attributed to rGO [38]. The peaks related to O–H groups in the GCN-5 sample are also noticed at around 3200 cm^−1^ due to the presence of a small percentage of rGO, which is shifted to lower wavenumber, indicating H-bonding interactions between CN-5 and rGO. The structural transformation of C_3_N_4_ nanosheets to QDs is also verified by UV–visible spectroscopy (Supporting Information File 1, Figure S4). The C_3_N_4_ nanosheet exhibits a sharp peak at ≈316 nm, which is shifted to ≈285 nm (higher energy) after the formation of QDs (CN-5 QD) due to the breaking of C=N links [39]. Hence, XRD, XPS, IR and UV studies show the successful structural transformation of g-C_3_N_4_ nanosheets to QDs and the presence of possible organic functionalities in the GCN-5.

**Figure 5:**
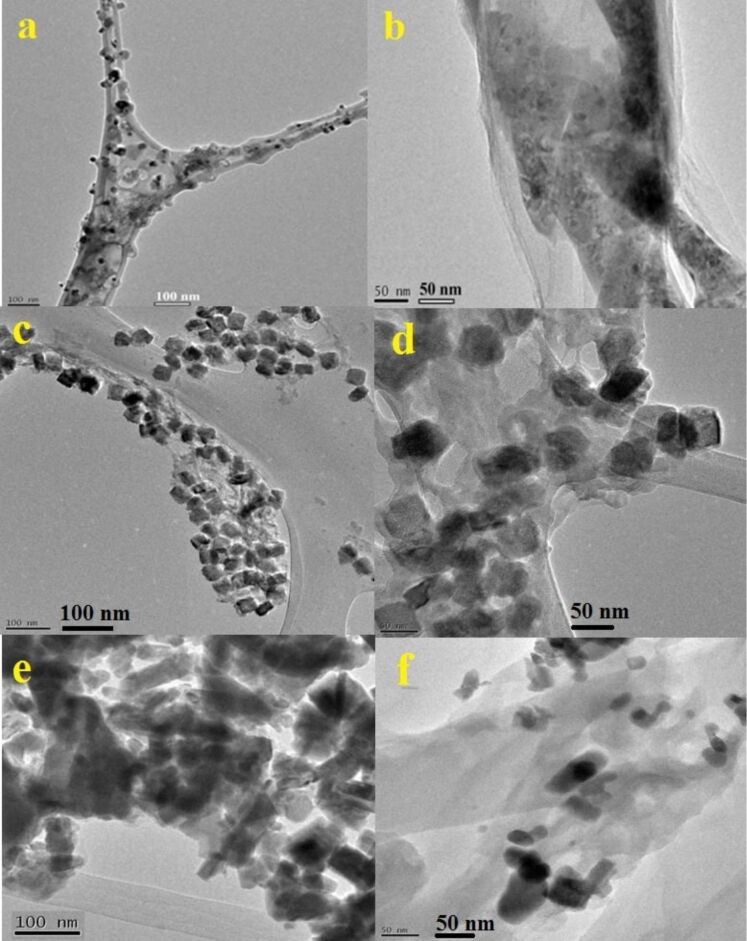
TEM images of the a) CN-5, b) GCN-5, c) CN-10, d) GCN-10, e) CN-20 and GCN-20 nanoflakes and quantum dots.

**Figure 6:**
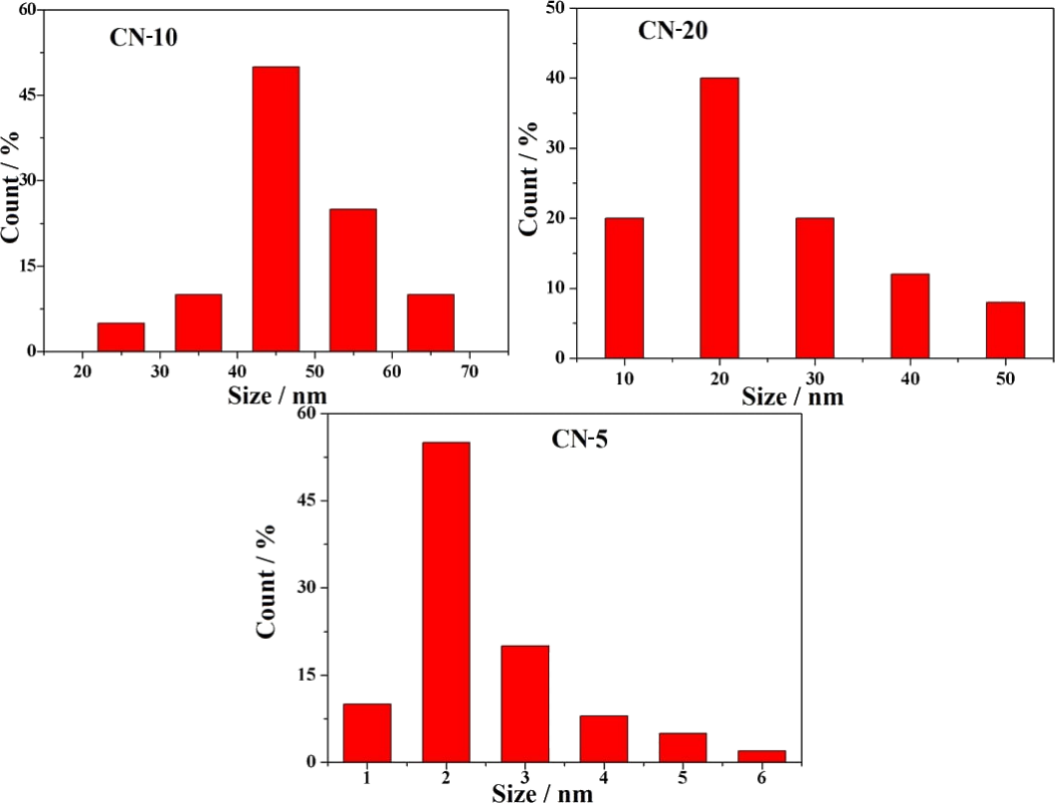
Particle size distribution of the synthesized CN nanoflakes and quantum dots.

The size, morphology, and distribution of the synthesized NFs and QDs were investigated by TEM and FESEM studies. Figure 5a,b shows TEM images of CN-5 and GCN-5 samples. It is observed that after the acid treatment and hydrothermal heating of g-C_3_N_4_ nanosheets at 190 °C for about 5 h, the g-C_3_N_4_ nanosheets are transformed into small nanoparticles along with QDs about 2–3 nm in diameter (Figure 5a). However, with a decrease in hydrothermal heating temperature (130 °C) and an increase in heating time (10 h and 20 h), the g-C_3_N_4_ nanosheets then produced a flake-like substance with an average size of 20–45 nm (Figure 5c and 5e). However, all particles are not completely transformed to QDs; there are also some larger particles along the QDs, as indicated by TEM. The TEM images clearly show (Figure 5b,d,f) that these NFs and QDs are well distributed and decorated with a thin rGO layer after the introduction of GO into the hybrid. Particle size distributions of the CN samples are also given in Figure 6. It is noticed that the average particle diameters of 45, 20, and 2 nm are obtained for CN-10, CN-20, and CN-5 samples, respectively. It is also observed that the size of the particles for 20 h heating (at 130 °C) is smaller compared to 10 h heating.

However, for 20 h of heating, some nanoflakes are found to be agglomerated, which is probably due to the extended heating time. However, for 10 h of heating, more uniform particles/flakes are observed as indicated by the TEM results. The TEM results suggest that the formation of QDs from g-C_3_N_4_ is temperature dependent. To further verify this, TEM images of the samples were taken at two different heating temperatures (150 and 170 °C) within the range 130 to 190 °C.

**Figure 7:**
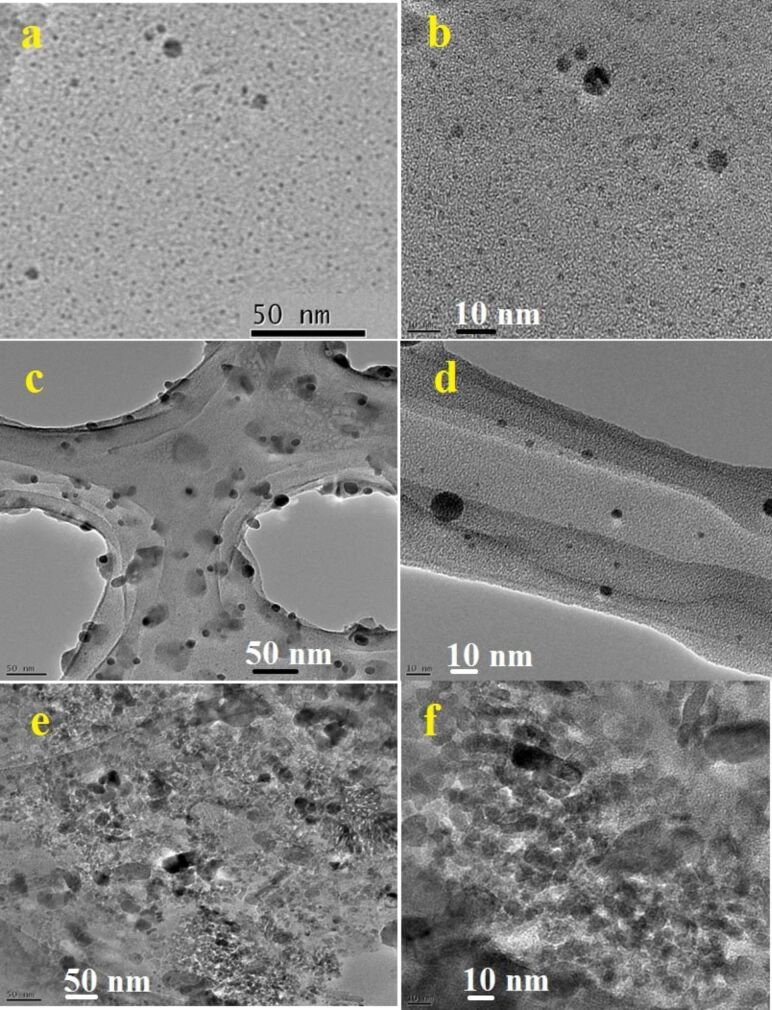
TEM image of CN nanoflake and quantum dot samples under heating at 190 °C for 5 h (a,b), 170 °C for 5 h (c,d), and 150 °C for 5 h (e,f).

The results confirm that the limiting temperature at which the nanoflakes are converted to quantum dots is around 170 °C (Figure 7). With an increase of temperature from 130 to 170 °C, the particle size starts to become smaller, and at above 170 °C, QDs are predominantly formed.

When a g-C_3_N_4_ nanosheet is subjected to acid etching followed by long term (10–20 h) hydrothermal heating at low temperature (130 °C), the g-C_3_N_4_ sheet breaks into pieces in different orientations and generates flake-like shapes of the material. Upon further increase of the heating temperature, the process proceeds very fast and cuts the flakes into dot-like structures (i.e., QDs) even within a small span of time (5 h). During the acid treatment process, some C–N bonds of the s-triazine units of the g-C_3_N_4_ sheet are oxidized and oxygen-containing carboxylate functional groups are generated at the edge and on the basal plane [19] as indicated by FTIR spectra (Figure 4). This results in orientational cleavage of g-C_3_N_4_ nanosheets and generation of some spherical particles of 300–500 nm as indicated by FESEM. Finally, CN QDs and NFs are formed after the hydrothermal treatment of these spherical particles at 130 to 190 °C. The surface of these CN QDs finally contains amino and carbonyl-functional groups, as indicated by XPS also.

**Figure 8:**
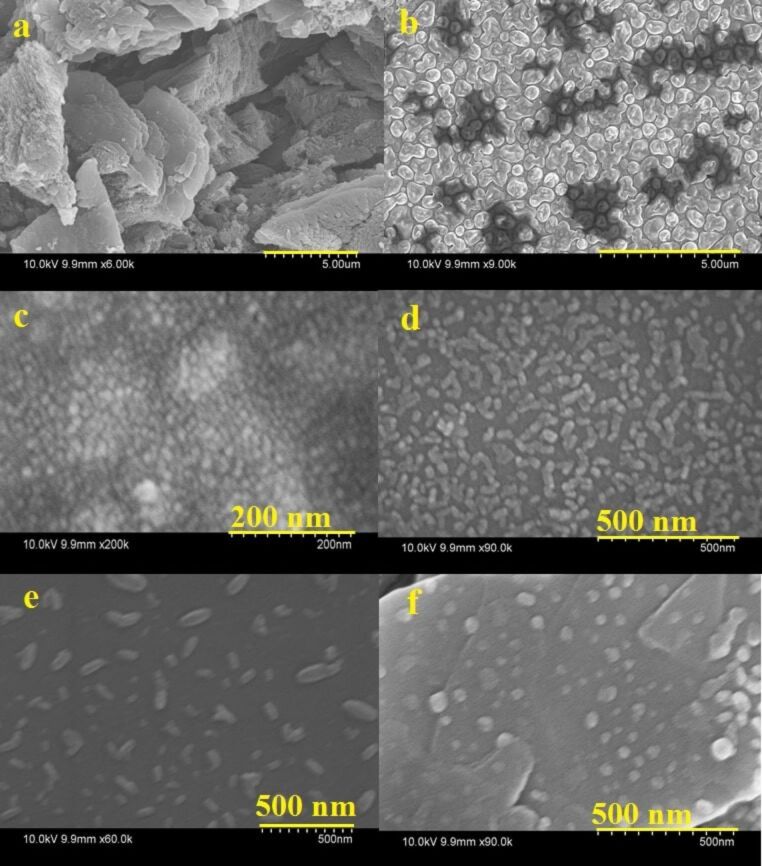
Field emission scanning electron microscopy images of the a) g-C_3_N_4_ nanosheet, b) after acid treatment of g-C_3_N_4_, c) CN-5 quantum dots, d) CN-10 nanoflakes, e) CN-20 nanoflakes and f) the GCN-5 material.

**Figure 9:**
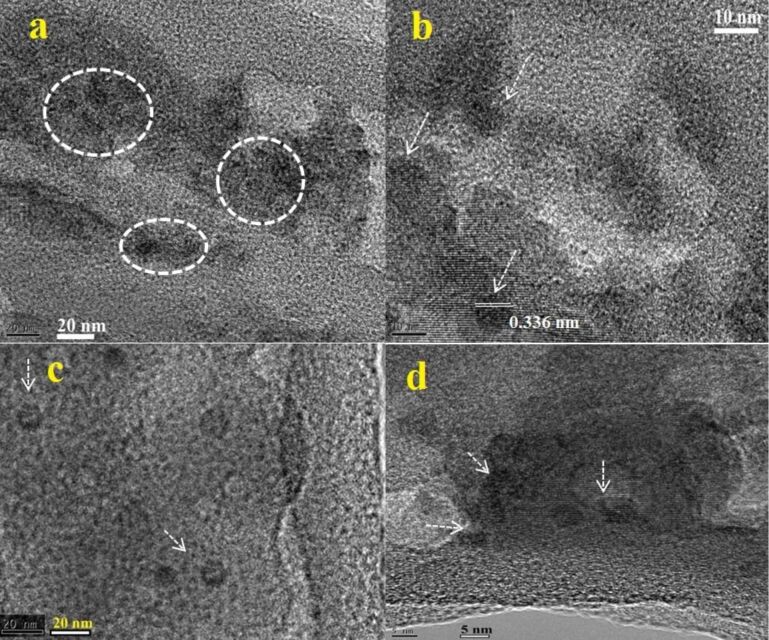
High-resolution transmission electron microscopy images of the GCN-5 quantum dots.

The TEM results are also in good agreement with the FESEM results. The FESEM image (Figure 8) clearly shows the structural transformation of g-C_3_N_4_ nanosheets into spherical particles with an average diameter of 300–500 nm after the acid treatment (Figure 8a and 8b). Figure 8c–e shows the FESEM images of the NFs and QDs (CN-5, CN-10 and CN-20). The flake-like morphology of CN-10 and CN-20 is clearly visible. The images of GCN-5 show that CN-5 QDs along with some larger nanoparticles of C_3_N_4_ are decorated on the rGO surface (Figure 8f); however, the exact size of the CN-5 QDs has been confirmed from the HRTEM results. Figure 9 exhibits HRTEM images of the GCN-5 QDs. It can clearly be seen that CN-5 QDs are decorated (marked by circle and arrows) onto the GO surface with an average diameter of 2–3 nm. A clear lattice spacing of 0.336 nm is also observed for the CN-5 QDs, which corresponds to the (002) plane of hexagonal g-C_3_N_4_, indicating crystalline nature of the QDs [40]. Hence, TEM, HRTEM, and FESEM studies confirm the morphology and size of the NFs and QDs and also confirm the presence of rGO in the hybrid material.

The band gaps of the prepared NFs and QDs are also calculated from UV–visible diffuse reflectance spectra (Supporting Information File 1, Figure S5), which are found to be 2.06, 2.15, 2.20, 2.22, 2.32 and 2.41 eV for the GCN-5, GCN-20, GCN-10, CN-5, CN-20 and CN-10 samples, respectively. The conduction and valence band potential of the GCN-5 sample are measured by using the Mulliken electronegativity theory [32]; *E*_CB_ = *X* − *E*^e^ − 0.5 *E*_g_, where *E*_CB_ is the CB edge potential; *X* is the geometric mean of the absolute electronegativity of the constituent atoms in the semiconductors, which is defined as arithmetic mean of the atomic electron affinity (*E*_EA_) and first ionization (*E*_ion_) energy; *E*^e^ is the energy of free electrons on the hydrogen scale (≈4.5 eV vs NHE); *E*_g_ is the band gap of semiconductors. The conduction and valence band potential value for GCN-5 are −1.01 and 1.05 eV, respectively, and is found to be lower than any CN NFs. For all other QDs/NFs and hybrid materials the CB values are also found to be more positive (lower) than g-C_3_N_4_ sheet [32].

### Photoreduction of CO_2_

**Figure 10:**
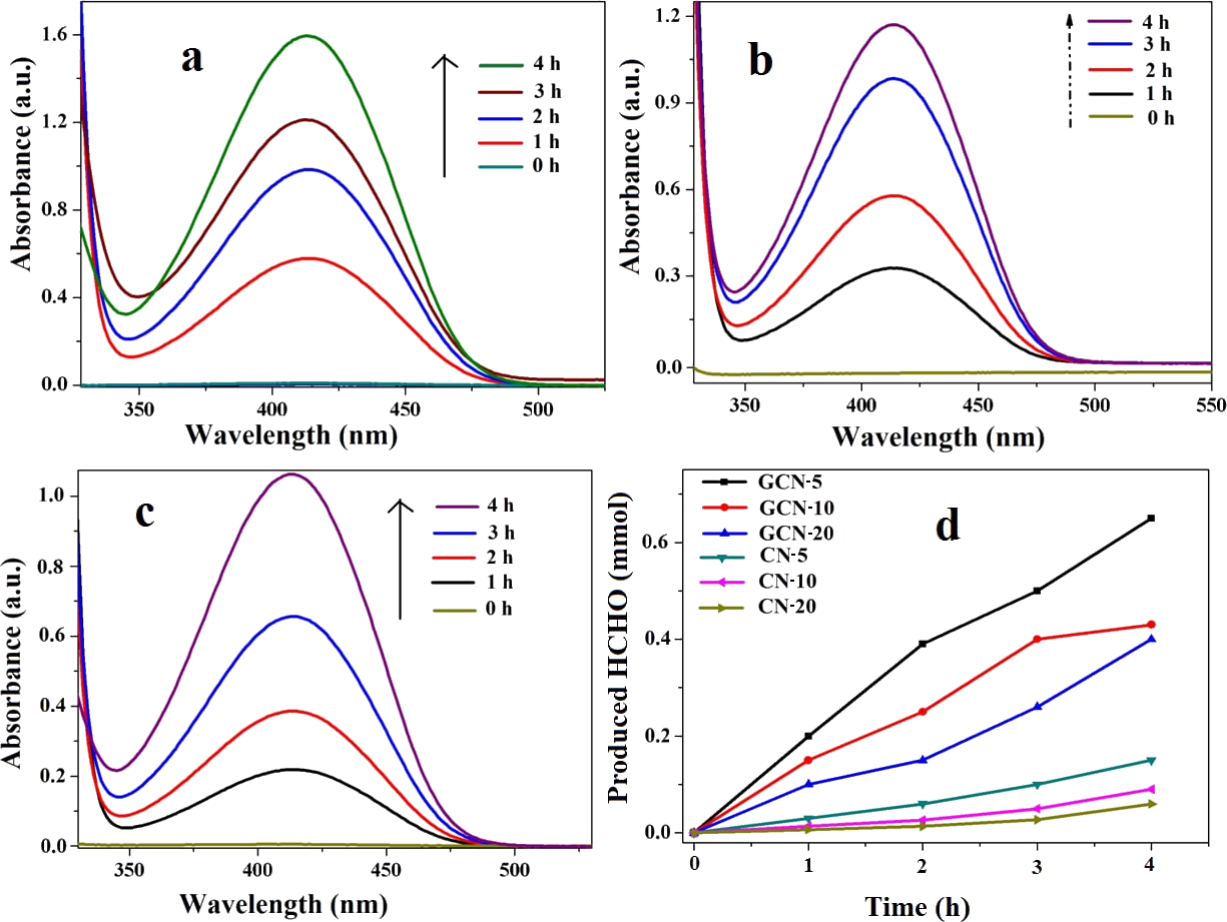
UV–vis absorption spectra of the photogenerated HCHO with Nash reagent (a–c), and (d) production rate of HCHO in the presence of different GCN photocatalysts.

For the photocatalytic reduction of CO_2_, catalytic amounts of GCN hybrid materials (0.5 g/L) are added to an aqueous Na_2_CO_3_ solution (0.01 M) in a round bottom flask capped with a rubber septum. The solution is then illuminated by 100 W halogen lamps. A sample (5 mL) of the photo-reacted solution is withdrawn after a measured interval and mixed with Nash’s reagent. The solution developed a bright yellow color due to the formation of a 3,5-diacetyl-1,4-dihydro-2,6-lutidine (DDL) complex between HCHO and the Nash reagent. The optical intensity of this complex is then measured at λ_max_ = 412 nm using a UV–vis spectrophotometer, and the final concentration of the produced HCHO was measured using a standard calibration curve (Supporting Information File 1, Figure S6). The UV–visible absorption spectra of the product used for the determination of HCHO in the presence of different GCN hybrids are shown in Figure 10.

**Figure 11:**
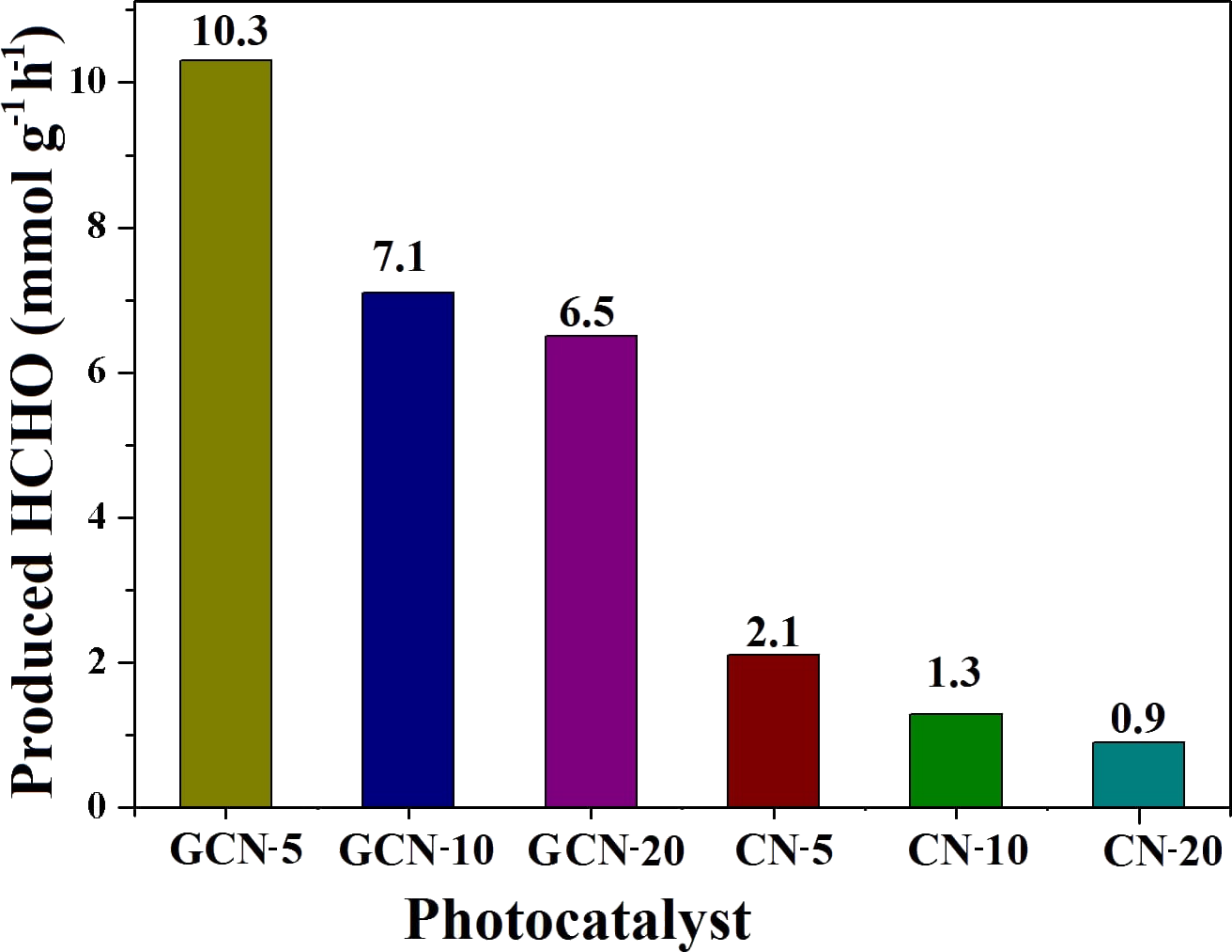
Apparent yield of HCHO in the presence of the various photocatalysts.

It is observed from the UV spectra that with increasing irradiation time, the peak of the DDL complex also increased due to effective production of HCHO. The production rates of HCHO for GCN-5, GCN-10, and GCN-20 are found to be 10.1, 7.1, and 6.5 mmol g^−1^ h^−1^, respectively (Figure 11). Compared to GCN-10 and GCN-20, GCN-5 exhibited the highest production of HCHO (680 μmol in 4 h) under visible light, and the photocatalytic efficiency of GCN-5 towards the reduction of CO_2_ is found to be better than many other catalysts reported previously in the literature [41–43]. The CO_2_ photoreduction efficiency of the GCN-5 sample was also measured by apparent quantum yield (AQY) measurements, and was found to be 22.3%. The AQY value of GCN-5 is higher than many earlier reports [44–46]. It is also clearly observed that in the presence of rGO, the photoactivity of CN-5, CN-10, and CN-20 catalysts is significantly enhanced (5 to 6 fold) for the production of HCHO, indicating that rGO provides a very active catalytic surface for the QDs and NFs, which increases the effective charge separation within the hybrids. Interestingly, the results also show that CN-5 exhibited almost two times higher reduction capacity of CO_2_ compared to CN-10 and CN-20. This is because of the QD size of CN-5, which increases its surface area (231.3 m^2^/g) compared to CN-10 (207.8 m^2^/g) and CN-20 (195.4 m^2^/g), thus increasing the number of catalytic active sites. The lower surface area of CN-20 was responsible for its poorer activity compared to CN-10, which is probably because of the agglomeration of CN-20, as suggested by TEM. The photoreduction ability of CN-5 is found to be higher than that previously reported for traditional C_3_N_4_ sheets [47–48].

We also carried out the reduction test with samples of different amounts of rGO from 2.5 to 7.0 mg with CN QDs (data not shown). However, the maximum activity is only observed for 5 mg rGO, and hence, this quantity was used for all cases in the present study. The higher loading of rGO yielded less activity, which is probably due to less light penetration in the sample since a higher concentration of rGO suspension blocks the effective light.

For a comparative study, the CN samples are also prepared at 130 °C for 5 h and at 190 °C for 10 h. The TEM study suggests (Supporting Information File 1, Figure S7) that there is no significant change in size of the CN QDs at 190 °C for 5 h and 10 h. The photo-reductive capacity of the CN at 190 °C for 10 h is also found to be almost identical to 190 °C for 5 h. This is possibly because of the similar size of the QDs formed at this temperature. However, for the CN sample at 130 °C of 5 h, it is observed that the size and morphology of the produced NFs are almost similar with CN-10, but a small portion of the CN nanosheet is still present with the NFs, indicating that there might be an incomplete transformation of the nanosheet to NFs. This is probably due to the reduced heating time at this low temperature; however, this did not affect the photoreduction capacity of these CN-NFs (130 °C of 5 h), which was found to be nearly similar to CN-10 (data not shown).

**Figure 12:**
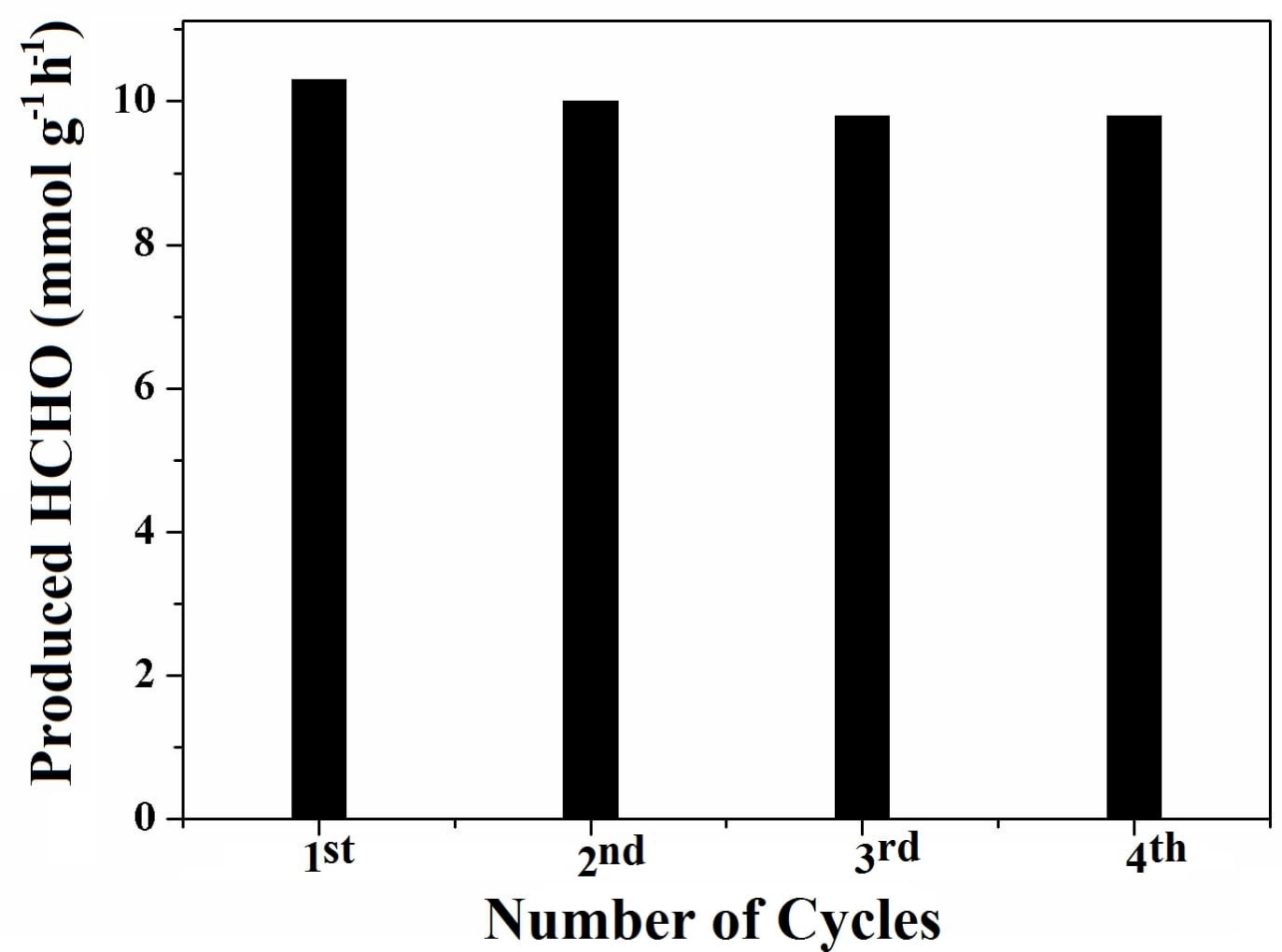
Photostability of the GCN-5 sample against the production of HCHO.

The stability of a catalyst is of paramount importance for practical applications. Hence, the prepared GCN-5 sample was recycled several times to check its reduction performance. It is observed that no significant loss in activity is observed even after the fourth cycle (Figure 12). Hence, the prepared metal-free GCN-5 is highly stable and can be reused for the photoreduction tests.

The photoreduction of CO_2_ to HCHO is also verified by gas chromatography mass spectrometry (GC–MS). A sample is examined for GC analysis after 4 h of photoirradiation with GCN-5 under identical conditions, and the spectrum shows a major peak related to HCHO and a minor peak for trace amounts of methanol, and no other peaks are found (Supporting Information File 1, Figure S8a), confirming the successful reduction of CO_2_ to HCHO.

Three sets of control experiments were also conducted, and an isotopic experiment to determine the source of carbon in the product: i) with Na_2_CO_3_ and GCN-5 under same condition, but without CO_2_, ii) absence of light under identical condition, iii) without GCN-5, and iv) an isotopic experiment under same conditions using ^13^CO_2_ (99.0% purity) and the irradiated sample are analyzed by GC–MS. In first three cases, no noticeable change in color is observed with Nash reagents, indicating no formation of HCHO. However, for the isotopic experiment, the GC–MS spectrum shows (Supporting Information File 1, Figure S8b) the major *m*/*z* peaks at 31 and 33, which are observed due to H^13^CHO, and ^13^CH_3_OH, respectively.

**Figure 13:**
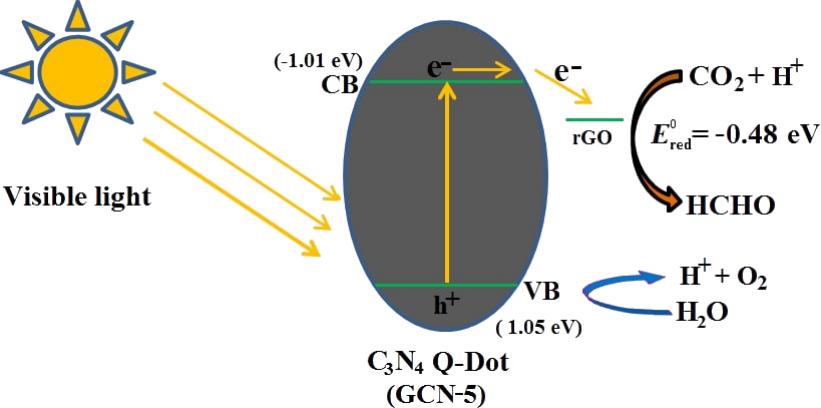
Possible photoreduction mechanism of CO_2_ to HCHO in the presence of the GCN-5 catalyst sample under visible light illumination.

The photoreduction mechanism of CO_2_ to HCHO in the presence of the GCN-5 catalyst under visible light illumination is shown in Figure 13. The CN QDs absorb visible wavelengths of sunlight due to their appropriate band edge potential value, thus exciting the electrons. These excited electrons are then transferred to the rGO surface, because it has a lower conduction band (CB) potential than CN-5, which improves the charge separation process in the GCN hybrid [28]. Finally, these electrons are utilized for the reduction of CO_2_ to HCHO in the presence of water reductant. At the valence band (VB), water is oxidized by photo-generated holes (h^+^), and generates protons (H^+^ ion), which initiate the CO_2_ reduction process. Since the reduction potential of CO_2_ to HCHO (*E*^0^_(CO2/HCHO)_ = −0.48 eV) matches with the CB potential of rGO, the CB electrons in rGO can easily reduce the CO_2_ [49] with the help of protons.

## Conclusion

Reduced graphene oxide supported C_3_N_4_ NF and QD hybrid (GCN) materials have been successfully synthesized via a sol–gel and hydrothermal method and are characterized in this work. The formation of g-C_3_N_4_ NFs (20–45 nm) and QDs (2–3 nm) can be controlled by varying the temperature during hydrothermal heating. XRD, TEM, IR, UV and XPS studies confirmed the structural transformation of the nanosheet to QDs, and also the presence of rGO in the GCN hybrid. The as-prepared CN-5 (QD) exhibited better photoreduction of CO_2_ and generation of HCHO compared to the CN-10 and CN-20 NFs due to its quantum size. Additionally, the production of HCHO is improved almost five-fold (10.3 mmol h^−1^ g^−1^) in the presence of rGO with CN-5. It is expected that the QD size of the CN-5 sample promotes a higher number of catalytic active sites and enhanced light absorption, and the rGO provides better charge separation, which further enhances its photo-reductive capacity. Therefore, metal-free GCN hybrid materials could be a potential candidate for CO_2_ photoreduction and HCHO (fuel) generation because of their excellent activity, stability and environmental sustainability.

## Experimental

### Materials

Urea (Duksan Pure Chemicals, South Korea), ammonium acetate (Duksan Pure Chemicals, South Korea), acetylacetone (Sigma-Aldrich), sodium carbonate, (Sigma-Aldrich), sodium nitrate (Sigma-Aldrich), and potassium permanganate (Tokyo Chemical Industry-TCI, Mark) were used as received. All other chemicals were of analytical grade (99.9%) and used without further purification.

### Preparation of g-C_3_N_4_ nanoflakes and quantum dots (CN-5, CN-10, and CN-20)

Bulk g-C_3_N_4_ was first prepared using urea as a precursor according to our previously described method [32]. In brief, an appropriate amount of urea (6.0 g) was placed in an alumina crucible and was calcined at 550 °C for 2 h at a rate of 5 °C/min under air. The as-prepared bulk g-C_3_N_4_ was further exfoliated by thermal oxidation at 500 °C for 2 h at a rate of 5 °C/min to obtain g-C_3_N_4_ nanosheets. The obtained 2D g-C_3_N_4_ nanosheets were then subjected to acid etching followed by hydrothermal treatment to obtain 0D g-C_3_N_4_ QDs [19]. In brief, the g-C_3_N_4_ nanosheets (0.5 g) were oxidized with concentrated H_2_SO_4_ (50 mL) and HNO_3_ (100 mL) for 4 h under ultrasonication. A clear solution was formed, which was diluted with deionized (DI) water (100 mL) to produce a cloud-like colloidal suspension of g-C_3_N_4_. The products were filtered through a 0.4 μm microporous membrane to remove the acid, carefully redispersed in deionized water under ultrasonication, and then transferred to an autoclave (90 mL) and heated at 130–190 °C for different times (5, 10, and 20 h) to yield g-C_3_N_4_ NFs and QDs. The product obtained after 5 h of heating at 190 °C is denoted as CN-5, and those obtained after 10 and 20 h heating at 130 °C are denoted as CN-10 and CN-20, respectively.

### Preparation of rGO@g-C_3_N_4_ nanoflake/quantum dot hybrid materials (GCN)

GO was synthesized by the modified Hummers’ method [50]. The as-prepared GO (5 mg) and CN NF/QDs (95 mg) were dispersed in 100 mL ethanol, and the mixture was sonicated for 30 min to form a homogeneous suspension. After that, the suspension was transferred into a 90 mL Teflon-lined autoclave and heated at 190 °C for 2 h. The resulting black rGO/g-C_3_N_4_ NFs/QDs (GCN) hybrid materials were collected by centrifugation and then washed with distilled water several times, and finally dried under vacuum at 50 °C. The final hybrid products are denoted as GCN-5, GCN-10, and GCN-20.

### Photocatalytic reduction of CO_2_

Photocatalytic reduction of CO_2_ was carried out by a spectrophotometric method [51]. GCN hybrid material (15 mg) was placed in 30 mL of an aqueous Na_2_CO_3_ solution (0.01 M) in a 50 mL two-necked round bottom (RB) flask which was sealed by a rubber septum. The solution was bubbled with CO_2_ (10 mL min^−1^) for 30 min to remove any dissolved oxygen. The reaction mixture was then illuminated by 100 W visible halogen lamps for 4 h. A sample (5 mL) of the mixture was collected every hour. The reduction rate was determined by measuring the concentration of the reduction product (i.e., formaldehyde), the amount of which was determined by a photospectrometric method using Nash’s reagent (prepared by 15 g ammonium acetate (2 M), 0.3 mL acetic acid (0.05 M), and 0.2 mL acetylacetone (0.02 M) in 100 mL water) [52]. The reagent was placed in the dark to avoid any photochemical reaction. The produced HCHO solution (0.5 mL) was then added to 5 mL of Nash’s reagent. Then the mixture was heated for 5–10 min in a water bath at 60 °C. The solution developed a bright yellow color, and the optical intensity was then measured at λ_max_ = 412 nm using a spectrophotometer. The concentration of produced HCHO was determined from a standard calibration curve.

The photoactivity usually depends on the quantity of catalyst and light intensity, therefore the CO_2_ photoreduction efficiency can also be measured by the apparent quantum yield (AQY), where AQY can be calculated according to Equation 1. The number of electrons required (4 e^−^) to obtain each HCHO is also considered, and the intensity of the light is measured by following standard potassium ferrioxalate method [53].

[1] $$\text{AQY}\left(\%\right)=\frac{4\times \text{number}\;\text{of}\;\text{produced}\;\text{HCHO}\;\text{molecules}}{\text{number}\;\text{of}\;\text{incident}\;\text{photons}}\times 100$$

### Characterization techniques

PXRD was carried out using an X’pert Pro PANalytical instrument (Cu Kα radiation, λ = 0.154 nm) within a 2θ range of 10–60°. XPS was conducted using a Thermo Kα XPS (Thermo Fisher Scientific). FESEM was carried out using an S-4800, Hitachi Ltd. instrument (acceleration voltage, 5 kV), and TEM was conducted with a Tecnai G^2^ F20 S-TWIN microscope. FTIR spectra were recorded with a Perkin Elmer Spectrum 100 spectrometer, and absorbance spectra were measured using a UV–visible spectrophotometer (Thermo Fisher Scientific). Gas chromatography coupled with mass spectrometry (GC–MS) was carried out on an Agilent 7890A instrument with both a thermal conductivity detector and a flame ionization detector and using helium as a carrier gas.

## Supporting Information

File 1 Additional figures.
