# Supplementary material for: Reduced graphene oxide supported C3N4 nanoflakes and quantum dots as metal-free catalysts for visible light assisted CO2 reduction
Source: Beilstein J Nanotechnol. 2019 Feb 13;10:448–58. doi: 10.3762/bjnano.10.44 (PMC6404395; doi:10.3762/bjnano.10.44)
Supplement: File 1 — Additional figures. [file Beilstein_J_Nanotechnol-10-448-s001.pdf]

BEILSTEIN JOURNAL OF NANOTECHNOLOGY

Supporting Information

for

Reduced graphene oxide supported C_3N_4 nanoflakes and quantum dots as metal-free catalysts for visible light assisted CO_2 reduction

Md Rakibuddin and Haekyoung Kim

Beilstein J. Nanotechnol. **2019,** *10,* 448–458. doi:10.3762/bjnano.10.44

Additional figures

License and Terms: This is a supporting information file under the terms of the Creative Commons Attribution License (http://creativecommons.org/licenses/by/4.0). Please note that the reuse, redistribution and reproduction in particular requires that the authors and source are credited.
The license is subject to the *Beilstein Journal of Nanotechnology* terms and conditions: (https://www.beilstein-journals.org/bjnano)

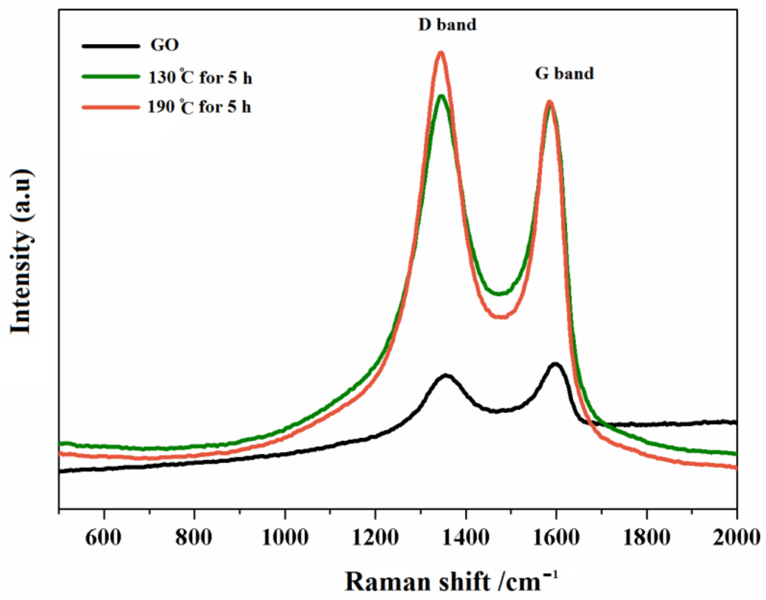

Figure S1: Raman spectra of GO samples at different heating temperatures.

The characteristic D band (1356 cm^{-1}), and G band (1597 cm^{-1}) of GO were blue shifted to 1345 cm^{-1} and 1586 cm^{-1}, respectively, after the hydrothermal treatment. The intensity ratio of the D and G bands, I_D/I_G, increases from 0.96 to 1.12 due to the increase in the number of isolated sp^2 domains. All these information indicates partial reduction of GO to RGO.

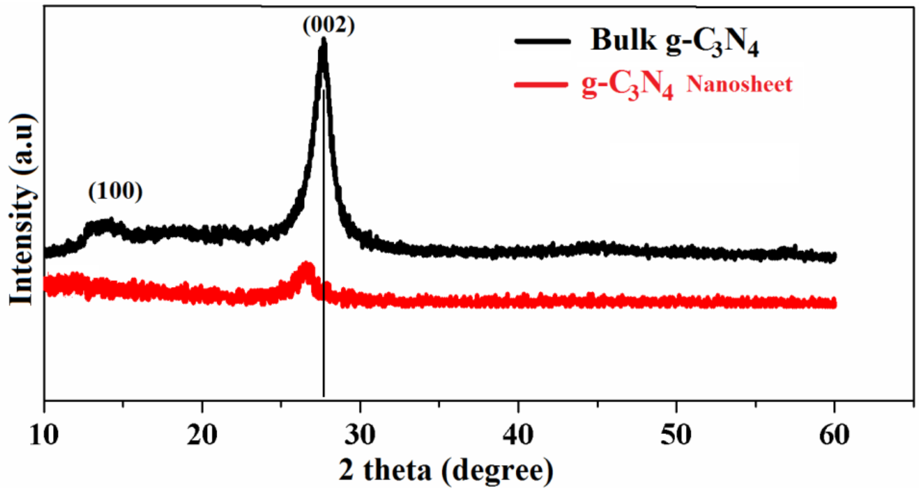

Figure S2: PXRD pattern of g–C_3N_4 bulk and nanosheet.

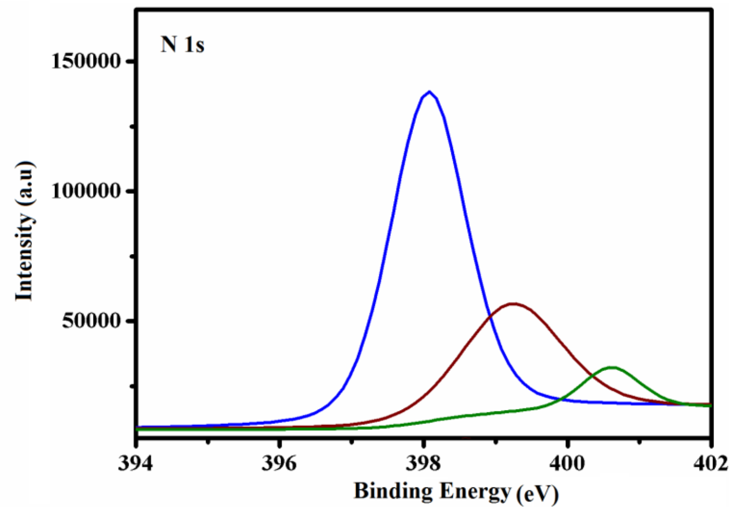

Figure S3: XPS fitting of N 1s of the pure g–C_3N_4.

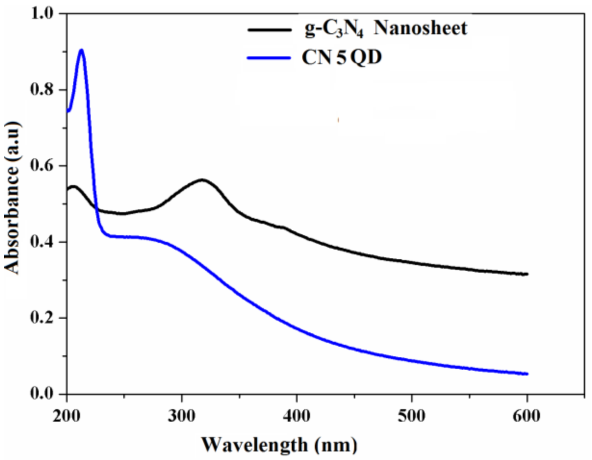

Figure S4: UV–visible spectra of the g–C_3N_4 nanosheet and CN-5 QD in water.

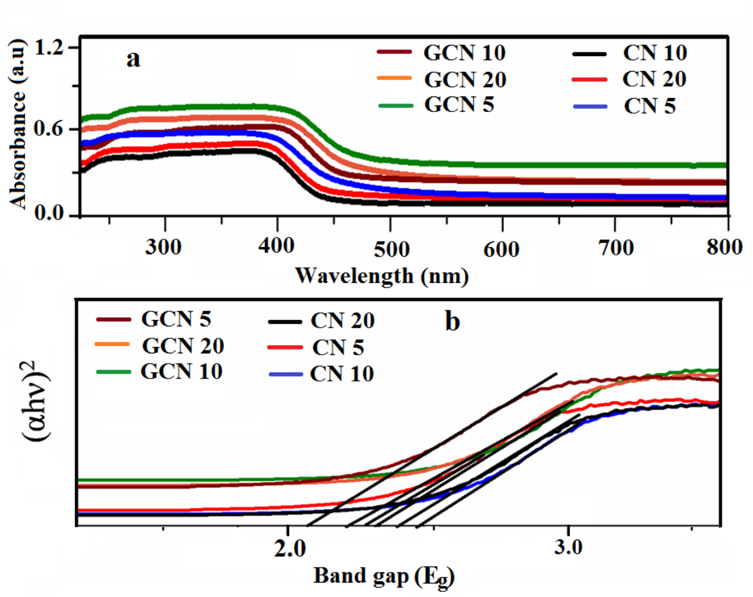

Figure S5: a) UV–visible spectra and b) Tauc plot of the CN and GCN NFs/QDs for determination of band gap.

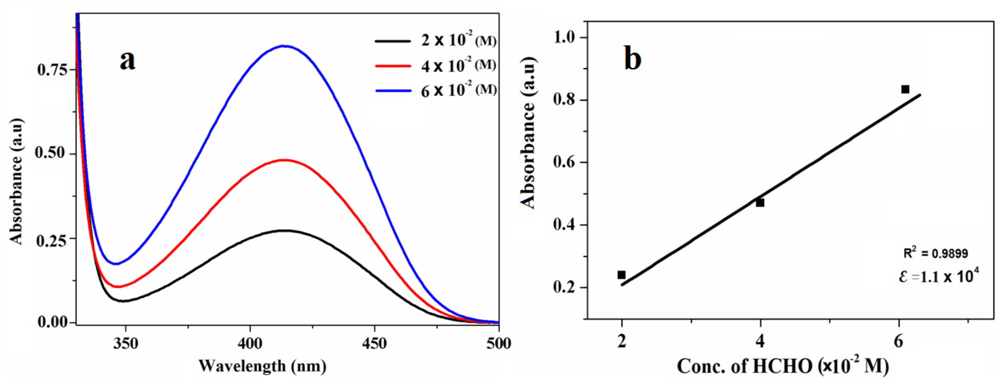

Figure S6: Standard calibration curve for determination of concentration of HCHO.

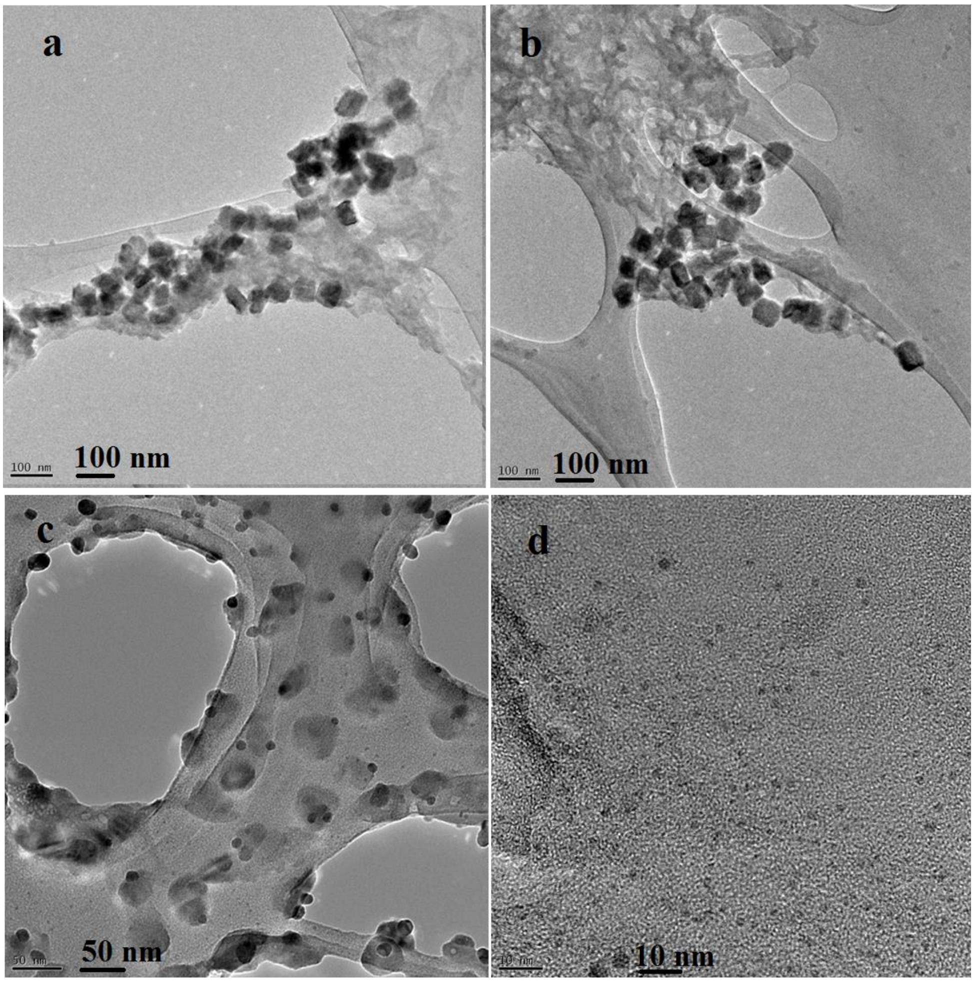

Figure S7: TEM image of the CN QDs at 130 °C for 5 h (a,b) and 190 °C for 10 h (c,d).

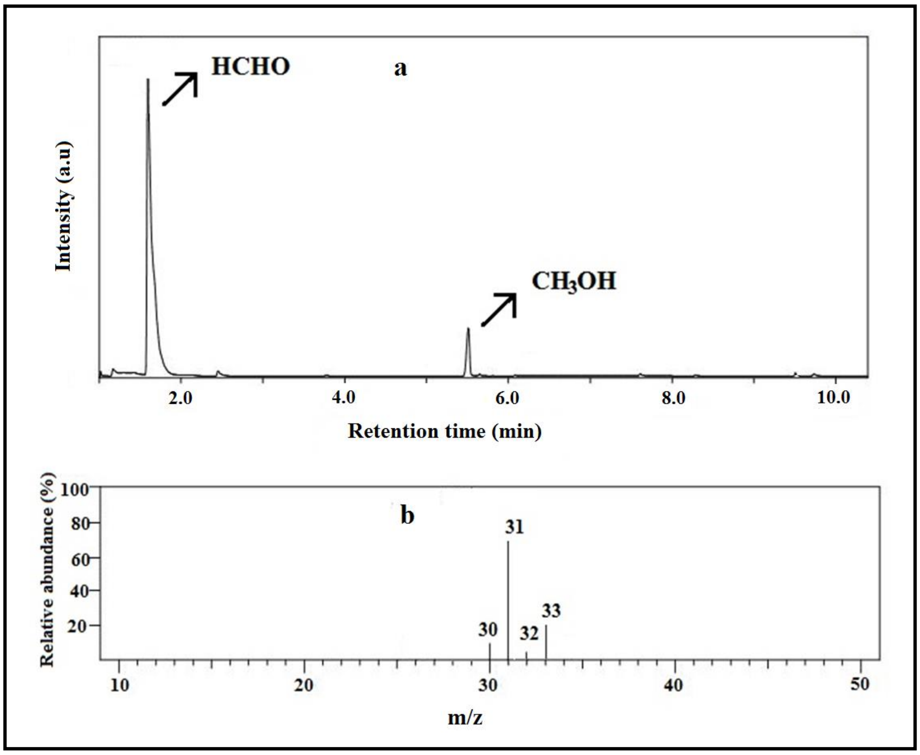

Figure S8: a) GC spectrum of the sample collected after 4 h of photoreaction in the presence of GCN-5, and b) GC-MS spectrum of photo-reacted sample treated with $^{13}CO_2$.
